# The placental pursuit for an adequate oxidant balance between the mother and the fetus

**DOI:** 10.3389/fphar.2014.00149

**Published:** 2014-06-24

**Authors:** Emilio A. Herrera, Bernardo Krause, German Ebensperger, Roberto V. Reyes, Paola Casanello, Mauro Parra-Cordero, Anibal J. Llanos

**Affiliations:** ^1^Laboratorio de Función y Reactividad Vascular, Programa de Fisiopatología, Instituto de Ciencias Biomédicas, Facultad de Medicina, Universidad de ChileSantiago, Chile; ^2^International Center for Andean Studies, Universidad de ChileSantiago, Chile; ^3^División de Obstetricia y Ginecología, Facultad de Medicina, Pontificia Universidad Católica de ChileSantiago, Chile; ^4^División de Pediatría, Facultad de Medicina, Pontificia Universidad Católica de ChileSantiago, Chile; ^5^Unidad Materno-Fetal, Hospital Clínico Universidad de Chile, Universidad de ChileSantiago, Chile

**Keywords:** hypoxia, oxidative stress, placenta, vascular dysfunction, high-altitude

## Abstract

The placenta is the exchange organ that regulates metabolic processes between the mother and her developing fetus. The adequate function of this organ is clearly vital for a physiologic gestational process and a healthy baby as final outcome. The umbilico-placental vasculature has the capacity to respond to variations in the materno-fetal milieu. Depending on the intensity and the extensity of the insult, these responses may be immediate-, mediate-, and long-lasting, deriving in potential morphostructural and functional changes later in life. These adjustments usually compensate the initial insults, but occasionally may switch to long-lasting remodeling and dysfunctional processes, arising maladaptation. One of the most challenging conditions in modern perinatology is hypoxia and oxidative stress during development, both disorders occurring in high-altitude and in low-altitude placental insufficiency. Hypoxia and oxidative stress may induce endothelial dysfunction and thus, reduction in the perfusion of the placenta and restriction in the fetal growth and development. This Review will focus on placental responses to hypoxic conditions, usually related with high-altitude and placental insufficiency, deriving in oxidative stress and vascular disorders, altering fetal and maternal health. Although day-to-day clinical practice, basic and clinical research are clearly providing evidence of the severe impact of oxygen deficiency and oxidative stress establishment during pregnancy, further research on umbilical and placental vascular function under these conditions is badly needed to clarify the myriad of questions still unsettled.

## INTRODUCTION

The placenta is the exchange organ between the pregnant woman and her developing fetus. The adequate function of this organ is clearly essential for a proper progress of gestation and a healthy baby as final outcome. As every developing organ, the placenta has an adapting capacity to variations in materno-fetal conditions, with short and long-lasting responses deriving in potential morphostructural and functional changes. These adjustments sometimes compensate the initial triggering insults and occasionally may switch to long-lasting remodeling processes arising maladaptation, affecting its own function and the materno-fetal health. Appropriate levels of oxygen and reactive oxygen species are determinants in placental development and function. This review will focus on placental responses to hypoxic environments, usually associated with oxidative stress and vascular functional impairments.

## HYPOXIC CONDITIONS DURING DEVELOPMENT

One of the most challenging conditions in modern perinatology is hypoxia during development. This is considered harmful to the fetus due to the short- and long-term devastating effects, particularly in the central nervous and cardiovascular systems, resulting in functional alterations. Furthermore, *in utero* adverse conditions can increase the risk of developing chronic diseases later in life, phenomena known as fetal programming or developmental origins of health and disease-DOHaD (Fowden and Forhead, 2009; Gunn and Bennet, 2009; Li et al., 2012). However, less focus has been put on the placenta and its protective role during development.

Hypoxia is defined as a deficient oxygen (O_2_) supply for the physiological demands of a tissue. This is a restrictive condition frequently faced during fetal life, either by maternal or umbilico-placental circumstances, or by environmental hypoxia as seen in highlands.

Nowadays, it is estimated that worldwide there are 150 million newborns per year and 5–10% of them will have low birth weight standardized for gestational age (Alberry and Soothill, 2007; Nardozza et al., 2012). Placental insufficiency leads to fetal growth restriction (IUGR) due to a decreased feto-placental perfusion and restricted oxygen delivery. This situation affects simultaneously O_2_ and nutrient supply to the fetus (Baschat, 2011), overlapping conditions that difficult the isolation of the specific effects of O_2_ deficiency in the determination of vascular impairment. Conversely, altitude decreases environmental pressure and consequently, alveolar PO_2_, deriving in high altitude hypoxia (Niermeyer et al., 2009). There is no precise data about the number and complications in high altitude pregnancies, but hypoxia-related problems during gestation are dramatically increased above 2500 m (Keyes et al., 2003; Moore et al., 2004; Zamudio, 2007; Julian, 2011). Currently, it is estimated that more than 150 million people live in highlands worldwide (Niermeyer et al., 2009). Of those, 35 million live in the Andean Mountains, in important cities, such as La Paz, capital of Bolivia (Keyes et al., 2003). This has led to an increased clinical and scientific interest in dissecting the different noxas that induce fetal programming (Luo et al., 2006; Franco et al., 2007; Nuyt, 2008; Schleithoff et al., 2012), but few have focus on the lack of oxygen as a main cause.

## HYPOXIA AND OXIDATIVE STRESS

**FIGURE 1:**
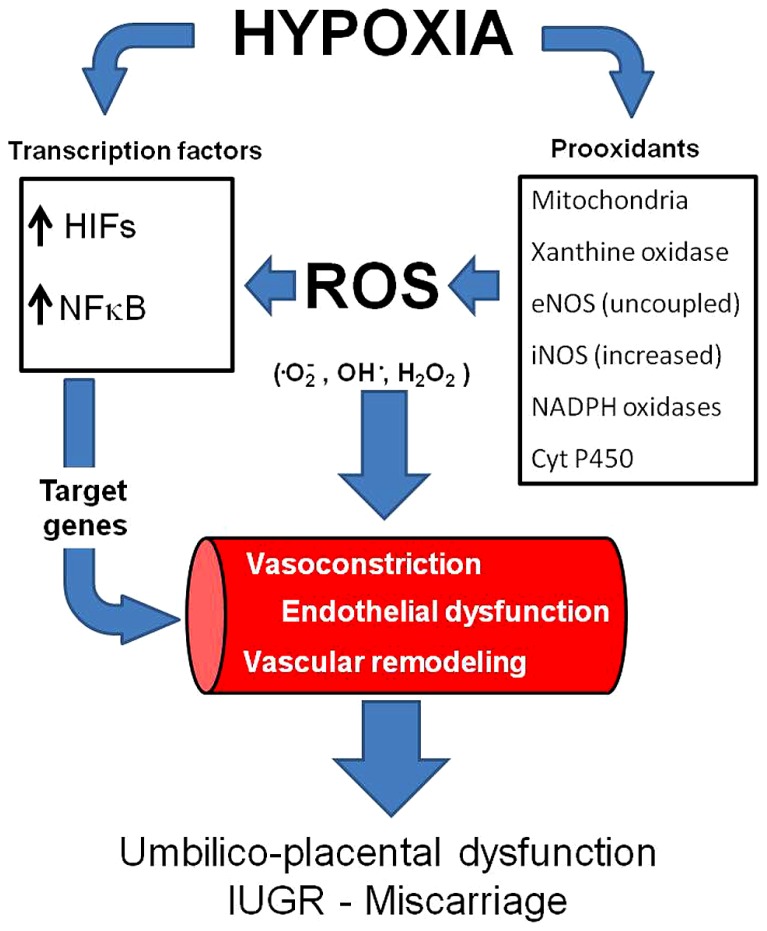
**Vascular responses induced by hypoxia and oxidative stress.** Hypoxia may increase ROS generation and induce (activate) transcription factors such as HIFs and NFκB. Consequently, the transcription of several genes is activated, inducing vasoconstriction and vascular remodeling. In addition, hypoxia and oxidative stress may induce endothelial dysfunction. All of the above impair vasodilatation capacity in the umbilico-placental vasculature and increases the risk of IUGR and miscarriage. ROS,: reactive oxygen species; HIFs, hypoxia inducible factors;, NFκB, nuclear factor-kappa B.

One of the mechanisms by which hypoxia induces damage is as a result of the increased generation of reactive oxygen species (ROS) by an incomplete reduction of oxygen (Abramov et al., 2007; Giussani et al., 2012; Pialoux and Mounier, 2012). ROS are free radicals produced as by-products of oxidation–reduction reactions. There are various intracellular enzymatic pathways that produce ROS in mammals, such as mitochondrial electron transport, NADPH oxidase, the cytochrome P450 monoxygenase system, xanthine oxidase, nitric oxide synthases, cyclooxygenase, and lipoxygenase (Apel and Hirt, 2004; Dowling and Simmons, 2009). Particularly in the vasculature, the main sources of superoxide anion (^•^O2-) are NADPH oxidase, xanthine oxidase, uncoupled endothelial nitric oxide synthase (eNOS) and mitochondria (Katusic, 1996; Förstermann and Sessa, 2012). Relatively low concentrations of ROS are necessary to operate as signaling molecules in the normal regulation of cell differentiation and function. However, the increased ROS generation may overwhelm the antioxidant endogenous capacity and determine oxidative stress (**Figure 1**). For instance, it has been established in several experimental models that ischemia/reperfusion, hypoxia/reoxygenation and even hypoxia itself can promote oxidative stress (Comhair and Erzurum, 2002; Guzy and Schumacker, 2006; Waypa et al., 2006; Behn et al., 2007; Clanton, 2007; Ward, 2007; Loor et al., 2011; Sylvester et al., 2012).

An important source of ROS is the mitochondrial electrons chain. At physiological oxygen levels, it has been suggested that about 1–4% of the O2 reduced by mitochondria may form ^•^O2- (Li and Shah, 2004). However, under conditions of metabolic perturbation such as hypoxia/reoxygenation (Pearlstein et al., 2002), the percentage of oxygen incompletely reduced can increase, promoting enhanced production of ROS by the mitochondria (Sanjuan-Pla et al., 2005; Guzy and Schumacker, 2006; Loor et al., 2011). Thus, studies have shown that a functional respiratory chain is required and that loss of cytochrome c (complex III), abolishes this ROS signal (Guzy et al., 2005; Guzy and Schumacker, 2006). Outstandingly, the direct addition of ROS such as H_2_O_2_ overcomes this blockade (Bell et al., 2007). These studies have led to the proposal that mitochondrial complex III is one of the main sources of ROS (Guzy and Schumacker, 2006).

In addition to ROS generated as a “byproduct” of cellular respiration, endogenous production of ^•^O2- also arises from NADPH oxidases (NOX 1, 2, 4, and 5) normally at low levels in smooth muscle and vascular endothelium (van der Vliet, 2008; Rivera et al., 2010). Another source of ROS is cytochrome P450, an enzyme involved in the metabolism of arachidonic acid in the vascular endothelium (Fleming et al., 2001). Superoxide anion and hydroxyl radical (^•^OH-) are produced during the cytochrome P450 reaction cycle when the electrons for the reduction of the central heme iron are transferred to the activated bound O_2_ molecule in an NADPH-dependent reaction (Fleming, 2001). Another important source of ROS is xanthine oxidase (XO), a metalloflavoprotein which is generated by the post-translational modification of xanthine dehydrogenase (XD) (Hewinson et al., 2004). Functionally, both XD and XO catalyze oxidation of hypoxanthine to xanthine and xanthine to uric acid, with the generation of ^•^O2- (Zhang et al., 1998; Meneshian and Bulkley, 2002). Endothelial nitric oxide synthase (eNOS) may also act as a generator of ROS and is dependent on NOS substrates and cofactors. Endothelial nitric oxide synthase is a calcium-dependent flavoenzyme that generates NO in a process that involves oxidation of the amino acid L-arginine via the reduction of O_2_ (Cai and Harrison, 2000). Intracellular calcium levels control the eNOS-calmodulin association which is an important stage in the activation of eNOS (Stuehr, 1999). Another protein which can control eNOS activity is heat shock protein 90 (HSP90), a molecular chaperone that increases eNOS activity, possibly by increasing the rate of electron transfer between the two domains of eNOS (Stuehr, 1999). The process of NO production also requires the essential NOS cofactor tetrahydrobiopterin (BH4) as it plays a crucial role in coupling the reduction of O_2_ to the oxidation of L-arginine as well as maintaining the stability of the NOS dimers (Vasquez-Vivar et al., 2003). If the production or efficiency of BH4 decreases, or if there is a deficiency in the NOS substrate L-arginine, then NOS can become uncoupled resulting in the production of ^•^O2- instead of NO (Lei et al., 2013; **Figure 1**).

### VASCULAR EFFECTS

Most studies on umbilico-placental vascular function are Doppler-based, therefore non-invasive and mostly present descriptive analysis. Nonetheless, *ex vivo* experimental approaches, such as vascular myography and placenta perfusion, have offered important clues of mechanisms modulating umbilico-placental vascular function in diverse perinatal conditions.

#### Vascular tone regulation

Vascular tone is dependent on the balance between vasodilator and vasoconstrictive agents. The endothelial-independent NO-induced vascular relaxation has been assessed in normal term placentas (Myatt, 1992; Wareing et al., 2005), and correlates inversely to Doppler pulsatility and resistance indexes (Wareing et al., 2005). Otherwise, in complicated pregnancies with fetal growth restriction umbilical artery pulsatility index inversely correlates with placental eNOS mRNA levels (Giannubilo et al., 2008). In addition, arginase, an endogenous negative regulator of eNOS which counteracts the NOS-dependent relaxation in umbilico-placental vessels (Krause et al., 2012), is increased by hypoxia in umbilical vein endothelium (Prieto et al., 2011) and in plasma from pre-eclamptic women (Sankaralingam et al., 2010). Furthermore, arginase inhibition restores the impaired NOS-induced relaxation in IUGR umbilical and chorionic arteries (Krause et al., 2013). Another vasodilator suggested to be involved in placental vasodilation is carbon monoxide (CO), which is decreased in preeclampsia and IUGR (Barber et al., 2001). In compensating CO and NO dysfunction in complicated pregnancies, an up-regulation of H2S has been shown to vasodilate the placental vasculature via potassium channels (Cindrova-Davies et al., 2013). Similar to other oxygen-sensitive vasculature, potassium channels are main determinants of vascular resistance (Wareing and Greenwood, 2011) and vasculogenesis (Brereton et al., 2013) in chorionic plate vessels. Previous studies have not been conclusive for the role of these channels in complicated pregnancies, but some authors suggests that K(V) may be involved in IUGR (Corcoran et al., 2008) and ROS might be regulating their function in the placenta (Mills et al., 2009), elevating vascular resistance. Furthermore, it has been shown that ROS enhanced basal tension and vasoconstriction in response to a thromboxane mimetic (Mills et al., 2009). Although the exact mechanism is unknown, K+ channels may be involved in maintaining a lower membrane potential, and therefore depolarization processes are easy to be activated and induce contraction. However, ROS may as well increase relaxation in response to NO (Mills et al., 2009).

The responses in placental vascular tone to different levels of oxygenation are still under debate and extensively reviewed by Wareing (2014). Some argue similar effects as those observed in the pulmonary bed, where oxygenation promotes vasodilation and others suggest a vasoconstriction response in umbilico-placental veins (Wareing, 2014). Even more, some authors sustain that vascular reactivity in chorionic arteries is independent of oxygen levels (Cooper et al., 2005; Wareing et al., 2006). The differences found might be dependent on the experimental conditions and the oxygenation levels, where much of these are made under relatively hyperoxic conditions when compared to the ranges of ~15–30 mmHg *in vivo* PO_2_ (Wareing, 2014). Furthermore, there is a differential response in the *ex vivo* experiments depending on the mode of delivery (Mills et al., 2007). Clearly the debate is open, and the responses in hypoxic pathological conditions cannot be predicted. Despite their fundamental importance for normal perfusion of the placenta, still the umbilico-placental vascular reactivity has been poorly studied, particularly in pathological conditions.

#### Vascular protein expression

The vascular effects on gene expression and cellular responses to hypoxia and oxidative stress are alike, sharing similar effector pathways. For instance, both conditions activate the hypoxia-inducible factor (HIF-1) and therefore induce several proteins associated with remodeling processes. It has been proposed that mitochondria are O_2_ sensors and signal HIF-1α stabilization by releasing ROS to the cytoplasm (Simon, 2006). HIF-1 is a transcription factor that is activated under hypoxia (Semenza and Wang, 1992) and regulates over 100 physiologically important genes that may affect the vascular function (Semenza, 2006). HIF-1α is in fact regulating the transcription of many vascular remodeling related genes (Yu et al., 1999; Schofield and Ratcliffe, 2004; Semenza, 2006), leading to increased tone, thickening and stiffening of the vasculature. Increases in HIF-1 and HIF-2 activity induce production of angiogenic growth factors and cytokines in hypoxic cells, such as VEGF, EPO, ANGPT, and PDGFB (Prabhakar and Semenza, 2012). All of these will induce vascular smooth muscle cell proliferation and a balanced tone towards increased resistance under chronic hypoxia (Prabhakar and Semenza, 2012). In addition, HIFs also play a more general role in the response to a variety of cellular activators and stressors, many of which use ROS as signal transducers (Cash et al., 2007; Görlach and Kietzmann, 2007). Although HIF-1 and HIF-2 may upregulate the expression of pro-angiogenic factors, the first tend to diminish the inflammatory response, whereas the second increases the response (Loboda et al., 2012). Therefore, in endothelial cells, both factors may have opposite effects, probably a fine tuning depending on the oxygen or ROS levels. HIF-3 is a third isoform, which has been less studied but postulated to act as a negative regulator of HIF-mediated transcription (Loboda et al., 2012). Currently, the most studied and with more ascribable roles in vascular expression responses if HIF-1. HIF-1α is increased in high-altitude placentas (Soleymanlou et al., 2005; Zamudio et al., 2007) and has an important role in lowland preeclampsia (Tal, 2012; Akhilesh et al., 2013), such as it has been proposed as a predictive biomarker of late pre-eclampsia (Akhilesh et al., 2013). A recent discussion has proposed that preeclampsia does not lead to hypoxia as has been presumed (Huppertz et al., 2014). In fact, Huppertz et al. (2014) cited three papers where the calculated PO_2_ seems to be higher in patients with preeclampsia and/or IUGR. However, no *in vivo* measurements have been ever done in uterine or placental tissue in complicated pregnancies, so the debate is still open.

Another transcription factor that is activated by hypoxia and oxidative stress is the nuclear factor kappaB (NF-κB) (Haddad, 2004). Although this factor is more related to innate immunity, inflammation, and apoptosis, it regulates genes that induce remodeling processes (Ungvari et al., 2006; Taylor and Cummins, 2009). Thus, NF-κB is involved in angiogenesis of the placenta (Min et al., 2003; Chen et al., 2008). The immune and inflammatory responses induced by NF-κB activation initiates and accelerates vascular remodeling, vascular inflammation, endothelium apoptosis, vascular oxidative stress and impaired NO bioavailability (Gao et al., 2008), which contribute to the blunted vascular function (Foncea et al., 2000; Csiszar et al., 2008). Furthermore, NF-κB may induce tumor necrosis factor-α (TNF-α), which plays a pivotal role in endothelial dysfunction (Gao et al., 2008; Zhang, 2008). It seems that NF-κB-induced local inflammatory reaction may have important placental dysfunctional features, at least in pre-eclampsia (Guedes-Martins et al., 2013). Recent studies have demonstrated that similar oxygen-sensing mechanisms, such as hydroxylases, are determinants on oxygen sensitivity for both HIF and NF-κB-dependent gene expression. Furthermore, there is an extensive degree of cross-talk occurring between NF-κB and HIF (Taylor and Cummins, 2009; Scholz and Taylor, 2013). Therefore, hypoxia and oxidative stress are activating transcription signaling pathways that may end in placental vascular dysfunctions (**Figure 1**).

## HYPOXIA, UMBILICO-PLACENTAL DEFICIENCIES, AND IUGR

**FIGURE 2:**
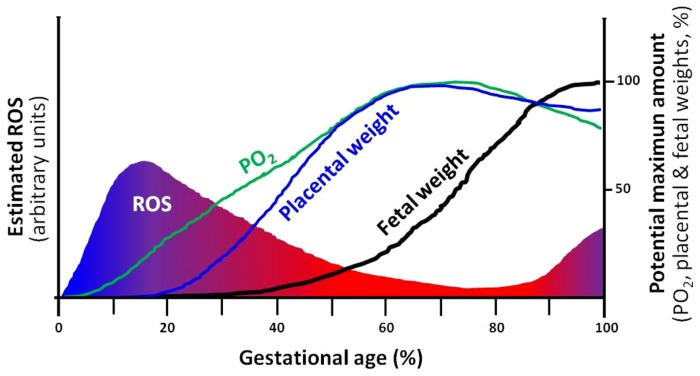
**Theoretical depiction proposing the correlation of ROS, PO_**2**_ and development of the placenta and the fetus.** The intrauterine (utero-placental) ROS formation is a function of PO_2_ in which, hypoxia supports elevations in ROS. Furthermore, the placental and fetal weights are closely related and dependent on increases in PO_2_ and limitation of ROS. Changes in any of the four variables, may directly affect the potential maximum amount of the rest of the variables. For instance, a decrease in PO_2_ may diminish fetal growth and term weight (IUGR). ROS, reactive oxygen species.

Hypoxia and oxidative stress levels in the placenta change along gestation. At initial stages of development, there is a reduced utero-placental vasculature and therefore, a low perfused and oxygenated environment. This hypoxic condition seems to be protective for the placenta and fetus as it is exposed to less oxygen radicals (Jauniaux et al., 2006). However, after trophoblastic invasion and placental development, the fetus and placenta demands for oxygen increases, and are covered by maternal blood supply in the intervillous space (Jauniaux et al., 2006; Schneider, 2011; Murray, 2012). In contrast, placental growth reaches its limits at term and terminal villi become over-crowded with diminished intervillous pore size. This physiologic condition will decrease the intervillous perfusion generating local hypoxia and oxidative stress (Schneider, 2011; Redman et al., 2014). Therefore, in the placental function, there is more chance of hypoxia and oxidative stress near delivery (**Figure 2**). Environmental hypoxic conditions or maternal problems that decrease PO_2_ may anticipate this physiological response to hypoxia in the placental unit. This condition is particularly important in the last trimester of gestation, when overlapping situations as placental and fetal energy demands are dramatically increased and the feto-placental unit respond in order to optimize the allocation of oxygen between competing demands (Schneider, 2011; Murray, 2012). As oxygen is a vital regulator of placental and fetal development (Genbacev et al., 1997; Zamudio et al., 2007), these responses are adaptive for fetal survival with evident restrictions in their demands, as seen in IUGR fetuses (Burton and Jauniaux, 2004; Soleymanlou et al., 2005; Soria et al., 2013).

Reduced fetal growth is well documented under the conditions of chronic hypoxia at high altitude (Keyes et al., 2003; Moore et al., 2004; Zamudio, 2007; Julian, 2011; Soria et al., 2013). In human high-altitude placentas, there are increased nitrotyrosine residues in the syncytiotrophoblast, nitrative stress and lower concentrations of anti-oxidants (Zamudio et al., 2007). These results show that hypoxia is inducing oxidative stress in placentas (Bosco et al., 2012). This is also observed in placentae from lowland women with pre-eclampsia and IUGR (Myatt et al., 2000). Further, maternal exposure to an equivalent level of chronic hypoxia in a rat model, significantly elevate maternal and placental molecular indices of oxidative stress, such as HSP70 and 4HNE (Richter et al., 2012). However, placentas at 3100 m do not increase total oxidative stress at delivery (Zamudio et al., 2007; Tissot van Patot et al., 2010). This was suggested to be a hypoxic preconditioning response that might occur in placentas that develop at high altitude (Tissot van Patot et al., 2010). The differences found in the placental level of oxidative stress might depend on the species, the period of exposure to hypoxia, the severity of the insult and the genetic background of the individual (Jauniaux et al., 2006; Richter et al., 2009, 2012; Tissot van Patot et al., 2010).

Another condition where placenta dysfunction and oxidative stress are directly involved is pre-eclampsia. Pre-eclampsia is a multi-system disorder characterized by high blood pressure and proteinuria in pregnant woman (Sibai, 2005). Although, no clear cause is known to induce this pathology, several proposals point toward an inadequate placentation and placental dysfunction (Jauniaux et al., 2006; Redman et al., 2014). Interestingly, high-altitude populations have increased incidence of pre-eclampsia (Escudero and Calle, 2006; Zamudio, 2007; Julian, 2011; Gonzales, 2012). In fact, this has been replicated in gestational hypoxia inducing preeclamptic-like symptoms in pregnant rats (Zhou et al., 2013). Furthermore, hypoxic stabilization of HIF-1α seems to have an important role in pre-eclampsia pathogenesis (Tal, 2012). Similar as in hypoxic conditions, pre-eclamptic placentas show increased oxidative stress (Vaughan and Walsh, 2012; Redman et al., 2014), potentially contributing to the impairment of placental perfusion by affecting vascular function (Myatt et al., 2000; Richter et al., 2009; Bosco et al., 2012). In pre-eclampsia there is a failure in trophoblast invasion and insufficient modification of spiral arteries, which has been described to lead, although not necessarily, to placental hypoxia (Huppertz et al., 2014). In addition to the functional impairment, the placental vascular bed is greatly reduced in chronic fetal hypoxia (Kuzmina et al., 2005; Sankar et al., 2013). The mechanisms involved will not only impact fetal life, but will have mediate- and long-lasting effects in the newborn (Gheorghe et al., 2007; Fowden and Forhead, 2009; Gunn and Bennet, 2009; Li et al., 2012; Giussani et al., 2012). The programming effects on the feto-placental unit due to intrauterine stress such as hypoxia and the potential mechanisms are currently intensively under research. The proposed main modulating mechanisms are epigenenetic, such as DNA methylation and histone deacetylation, reviewed in this issue by Casanello et al. (2014).

Clearly, the placental and fetal developments are dependent on oxygen availability and ROS restraint. Moreover, the levels of oxygen and ROS are as well dependent on placental function, creating interdependence among these variables and fetal development and growth (**Figure 2**).

## CLINICAL EVIDENCE

The combination of fetal biometry and maternal and fetal Doppler provides the best clinical approach, which are complementary to each other to identify small for gestational age fetuses (SGA) as well as those with intrauterine growth restriction (IUGR) at risk of adverse outcomes due to placental insufficiency (Figueras and Gardosi, 2011; Parra-Saavedra et al., 2014). Although the precise mechanisms by which placental function is affected remain unknown, there may possibly be a primary defect in placental development as the underlying abnormality. For the vast majority of the IUGR cases, the underlying defect is due to poor trophoblastic invasion of maternal spiral arteries and reduced uteroplacental flow (Brosens et al., 2011; Everett and Lees, 2012). When oxygen delivery to the fetus falls below a critical value, fetal oxygen uptake and glucose transfer are reduced and fetal hypoglycemia leads to gluconeogenesis from hepatic glycogen stores (Economides and Nicolaides, 1989; Nicolini et al., 1989). There is a down-regulation of active placental transport, independent of the presence of hypoxia or the severity of the IUGR and the fetus needs to mobilize other energy sources, resulting in more widespread metabolic changes such as alteration of amino acid metabolism and transport across the placenta (Cetin and Alvino, 2009; Avagliano et al., 2012). Maternal metabolic signals (leptin, insulin, and adiponectin plasma levels) lead to placental regulation of nutrient transport to the fetus (Lager and Powell, 2012) An increasing degrees of fetal metabolic compromise has been documented by cordocentesis in human fetuses showing that some SGA fetuses are hypoxemic, hypercapnic, hyperlacticemic, acidemic, and hypoaminoacidemic (Cetin and Alvino, 2009; Avagliano et al., 2012). In umbilical venous blood, mild hypoxemia may be present in the absence of hypercapnia or acidemia. However, in severe utero-placental insufficiency the fetus cannot compensate hemodynamically and thus, hypercapnia and acidemia increase exponentially. The immediate effect of decreased fetal glucose and amino acids levels is the down-regulation of the principal endocrine growth axis involving insulin, IGF I, IGF II and transforming growth factor beta adding negative impact on fetal growth (Randhawa, 2008; Lager and Powell, 2012).

The endothelial regulation of umbilico-placental vascular tone may be abnormal in pregnancies complicated by IUGR. Although the mechanisms may be diverse in the vascular dysfunction, hypoxia and oxidative stress play critical roles. Prostacyclin release during cordocentesis is decreased in pregnancies complicated by IUGR (Rizzo et al., 1996). Elevated levels of ET-1 were found in samples of umbilical venous blood obtained from IUGR pregnancies with abnormal Doppler waveforms (Erdem et al., 2003) and the villous core receptors for ET-1 appear to be functional. On the other hand, nitric oxide is the main vasodilator of the uteroplacental blood flow and it has also been suggested that inhibition of its synthesis reduces basal perfusion and increases flow resistance (Myatt et al., 1997; Parra et al., 2001). However, it has been postulated that the marked hypoxic vasoconstrictive response in pathological pregnancies is mediated by inhibition of potassium channels in small intraplacental vessels (Hampl et al., 2002). Hypoxemic growth-restricted fetuses also show a whole range of haematological abnormalities, including erythroblastemia and thrombocytopenia. Hypoxia is a trigger for erythropoietin release and stimulation of red blood cell production, through both medullary and extramedullary sites, resulting in polycythemia (Pallotto and Kilbride, 2006). Furthermore, there is a reduction in platelet count which inversely correlates with umbilical artery Doppler, and can be explained by placental consumption or dysfunctional erythropoiesis and thrombopoiesis (Baschat et al., 2013).

In IUGR pregnancies, histological studies have shown that the process of spiral artery vascular transformation is incomplete. In a normal pregnancy, the luminal diameter of the spiral arteries is greatly enlarged, and the walls are remodeled such that they contain very little smooth muscle. These changes extend into the vessels to the inner third of the myometrium to provide a low-resistance circuit for perfusion of the intervillous space. These modifications are associated with endovascular invasion of the fetal trophoblast into these maternal vessels. In women with IUGR, endovascular invasion and spiral artery remodeling occur either very superficially or they do not occur, being associated with high resistance to flow in the maternal uterine arteries and relative placental hypoperfusion (Brosens et al., 2011; Everett and Lees, 2012). Increased impedance in the fetal umbilical arteries becomes evident only when at least 60% of the placental vascular bed is obliterated (Chaddha et al., 2004). In pregnancies with IUGR, those with absent end-diastolic frequencies, compared to those with normal Doppler, have more fetal stem vessels with medial hyperplasia and luminal obliteration, and those with reversed end-diastolic flow have more poorly vascularized terminal villi, villous stromal haemorrhage, “haemorrhagic endovasculitis,” and abnormally thin-walled fetal stem vessels (Salafia et al., 1997). Absent/reversed end diastolic flow in umbilical artery represents the extreme end of the spectrum and this finding is associated with a high perinatal mortality, particularly after 30 weeks of pregnancy (Parra-Saavedra et al., 2014).

In fetal hypoxemia there is an increase in the blood supply to the brain and reduction in the perfusion of the kidneys, gastro-intestinal tract and the lower extremities (the so-called brain sparing effect; Baschat, 2004). Although knowledge of the factors governing circulatory readjustments and their mechanism of action is incomplete, it appears that partial pressures of oxygen and carbon dioxide play a role, presumably through their action on chemoreceptors. This mechanism allows preferential delivery of nutrients and oxygen to vital organs, thereby compensating for diminished placental resources (Vyas et al., 1990). On the other hand, the hemodynamic changes occurring in fetal arterial vessels during hypoxaemia and acidaemia induced by uteroplacental insufficiency are vasoconstriction, expressing as increased impedance to flow in descending aorta and renal artery (Griffin et al., 1984). Therefore, as a consequence of the brain sparing condition, there are selective modifications in cardiac afterload with a decreased left ventricle afterload due to cerebral vasodilatation, and an increased right ventricle afterload due to the systemic and pulmonary vasoconstriction (Groenenberg et al., 1989). Furthermore, hypoxemia may impair myocardial contractility while the polycythemia, which is usually present, may alter blood viscosity and therefore preload (Soothill et al., 1987). Alterations in fetal venous circulation will manifest an increase of reverse flow in the inferior vena cava during atrial contraction, and progressive fetal deterioration, suggesting a higher pressure gradient in the right atrium (Hecher and Hackelöer, 1997). The next stage of this disease is the extension of these abnormal blood velocities to the ductus venosus, rising systolic pressure and reducing diastolic filling as an expression of myocardiac dysfunction (Baschat, 2004). Finally the high venous pressure induces a reduction of velocity at the end of diastole in the umbilical vein, causing typical end diastolic pulsations (Rizzo et al., 1995). The epilogue of these clinical circumstances is cardiovascular impairment and umbilico-placental dysfunction.

## CONCLUSION

The full understanding of the umbilico-placental vascular functions will have important implications in developing therapies for oxidative-stress and hypoxia complicated pregnancies. Oxidative stress in chronic hypoxic conditions during gestation arises in multiple organ systems and subcellular compartments. This occurs due to an imbalance between cellular pro-oxidant and/or anti-oxidant detoxifying mechanisms. The umbilico-placental vasculature during pregnancy seems to be particularly affected as highly sensitive territories to changes in levels of oxygen and reactive oxygen species. Although these aspects have suggested the rationale for antioxidant therapy during pregnancy, still there are no effective treatments. Several randomized controlled trials have been performed to determine whether antioxidants supplementation in complicated pregnancies are beneficial, showing no evidence that these supplements may prevent preeclampsia (Poston et al., 2006, 2011; Basaran et al., 2010; Roberts et al., 2010; Xu et al., 2010; Conde-Agudelo et al., 2011; Kalpdev et al., 2011; Rumbold et al., 2011; Polyzos et al., 2012). In marked contrast, animal models and few human experiences have shown effectiveness in the use of different antioxidants preventing pregnancy complications and short- and long-term vascular dysfunction during pathologic pregnancies (Richter et al., 2009; Tara et al., 2010; Parraguez et al., 2011; Giussani et al., 2012; Wibowo et al., 2012; Jones et al., 2013; Kane et al., 2013). Nowadays, it is not recommended the use of antioxidants in pregnancy but the debate is still open and it merits new therapeutical approaches as it is clear that oxidative stress is partly determining complications. Further research on umbilical and placental vascular function under stressed conditions is still required.

## Conflict of Interest Statement

The authors declare that the research was conducted in the absence of any commercial or financial relationships that could be construed as a potential conflict of interest.

## ABBREVIATIONS

4HNE: 4-Hydroxynonenal
ADMA: asymmetric dimethylarginine
ANGPT: angiopoietins
BH4: tetrahydrobiopterin
CO: carbon monoxide
DOHaD: developmental origins of health and disease
eNOS: endothelial nitric oxide synthase
EPO: erythropoietin
ET-1: endothelin-1
GPx: glutathione peroxidase
H_2_O_2_: hydrogen peroxide
HO: heme oxygenase
HIF: hypoxia-inducible factor
HSP70: heat-shock protein 70
IGF: insulin-like growth factor
IUGR: intrauterine growth restriction
K(V): voltage-gated potassium channel
K+: potassium
NADPH: nicotinamide adenine dinucleotide phosphate
NF-κB: nuclear factor kappaB
NO: nitric oxide
O_2_: oxygen
^•^O2-: superoxide anion
^•^OH-: hydroxyl radical
PDGFB: platelet-derived growth factor subunit B
PE: preeclampsia
ROS: reactive oxygen species
SGA: small for gestational age fetus
SOD: superoxide dismutase
TNF-α: tumor necrosis factor-α
VEGF: vascular endothelial growth factor
XD: xanthine dehydrogenase
XO: xanthine oxidase.
